# Visual impairment and its associated factors among medical and health sciences students at the University of Gondar, Northwest Ethiopia

**DOI:** 10.1371/journal.pone.0255369

**Published:** 2021-08-19

**Authors:** Mihret Getnet, Yonas Akalu, Baye Dagnew, Yibeltal Yismaw Gela, Yitayeh Belsti, Mengistie Diress, Sofonias Addis Fekadu, Mohammed Abdu Seid

**Affiliations:** 1 Department of Human Physiology, School of Medicine, College of Medicine and Health Sciences, University of Gondar, Gondar, Ethiopia; 2 Department of Optometry, College of Medicine and Health Sciences, University of Gondar, Gondar, Ethiopia; 3 Department of Biomedical Sciences, Unit of Human Physiology, College of Health Science, Debre Tabor University, Debre Tabor, Ethiopia; King Saud University, SAUDI ARABIA

## Abstract

**Background:**

Visual impairment (VI) is a decreased ability to see things which is critical problem for health professionals and students to whom normal vision is essential for their daily activity. If it is not timely managed, visual impairment leads to physical, psychological, and socio-economic malfunctions to the victims and nation. Despite the above impacts, currently there is no study in Ethiopia on this topic. Therefore, this study aimed to determine the prevalence of visual impairment and its associated factors among Medical and Health Science students at the University of Gondar.

**Methods:**

A cross-sectional study was conducted from January to March 2020 among Medical and Health Science students. After recruiting study participants using systematic random sampling technique, we applied pre-tested self-administered questionnaire for sociodemographic variables and also visual acuity measurement was performed using Snellen chart placed at a distance of 6 meters. Visual acuity tested separately for each eye and for both eyes in a well- illuminated area. Pinhole was used for those participants with a visual acuity of < 6/9. Participants with a presenting visual acuity of < 6/12 in the better eye were diagnosed as having visual impairment. Epi data version 3.1 and stata version 14 were used for data entry and statistical analysis, respectively. Binary logistic regression was used, and the Adjusted Odds Ratio (AOR) with the 95% Confidence Interval (CI) was reported to declare the statistical significance and strength of association between VI and independent variables.

**Results:**

A total of 654 students were screened for visual acuity. The prevalence of visual impairment was 12.5% (95% CI: 10.21, 15.31). Age above 25 years (AOR = 1.8; 95% CI: 1.02, 3.26) and current alcohol drinking (AOR = 2.9; 95% CI: 1.7, 5.00), were statistically significant factors of visual impairment.

**Conclusion:**

The prevalence of visual impairment among medical and health science students was high. Age of study participant and current alcohol consumption were statically significant factors. This study warrants the routine screening of Medical and Health science students for visual impairment.

## Introduction

Visual system is one of our most important sensory systems mainly used for integration between individuals and the external environments by developing vision from the entry of light into the eye and the perception of this stimulus by the occipital lobe of the brain [1].

Visual impairment (VA) is a major health problem all over the world [2,3], which is characterized by a presenting visual acuity (VA) of less than 6/12 and of low vision less than 6/18, but equal to or better than 3/60, or a subsequent loss of visual field less than 20 degrees with the best possible correction in the better eye [4].

As estimated by the Lancet Global Health Commission, 1.1 billion people have impaired vision worldwide and the incidence is increasing [5]. It has been estimated that 75–90% of all teaching in the classroom comes either entirely or partially from the visual pathway to the students [6,7].

It has considerable social, psychological, and economic consequences for the patients and their caregivers. Employment in certain occupations such as working in the capacity of pilots, drivers, and a few others often requires a normal vision and hence visually defected students are likely to be removed from their professional work [8–10]. Cataract, refractive error, and trachomatous corneal opacity are the main causes of low vision and blindness globally within the general population [3].

Despite the main causes of visual impairment are either preventable or treatable [11,12], the burden of visual impairment is not distributed equally all over the world. Around 90% of people with visually impairments are living in developing countries [13]. Three quarters of the world’s blind children live in the poorest countries of Africa and Asia [14]. More than 300,000 out of the 1.4 million blind children worldwide live in Africa. In a nation, the prevalence of blindness was related to the dietary, health, and socioeconomic status of that country [15]. The prevalence of bilateral reduced visual acuity (VA < 6/12 in the better eye) was 0.7% [16], in a study conducted on rural primary school children in Tanzania.

There are an estimated 6 million blind people in Sub-Saharan Africa and 16–18 million people with low vision. Around 60% live in 20 African countries, including Ethiopia [11,17].

The eye problem in Ethiopia is among the main public health issues. It has enormous economic and social impacts for the affected person, the society, and the nation at large [18].

The prevalence of low vision in Ethiopia is 3.7 percent with major regional differences. The significant proportion of this issue (91.2%) is due to removable (either preventable or treatable) causes [19]. Nonetheless, if it is not detected early, it may cause permanent blindness. The adverse effect is felt over the remaining years of life when visual loss is present at a young age [10].

Moreover, visual impairment in adults have consequential impact on academic achievement and other day to day activities, such as ability to participate in physical exercise safely [20]. Poor academic performance affects self-confidence of the students and their future careers after graduation [21]. Even though there are some studies conducted in primary school students to assess the prevalence of VI, no one gives attention for adults joining higher education. Therefore, the main aim of this study was to determine the prevalence of visual impairment and its associated factors among medical and health science students at University of Gondar, Gondar, Ethiopia.

## Methods and materials

### Study setting and population

An institutional based cross-sectional study was conducted at University of Gondar College of medicine and health science, in Gondar city, which is located 727 km Northwest of Addis Ababa, capital city of Ethiopia. University of Gondar was established in 1954 as a college of public health, and currently it has 2,546 academic staff and 45,000 undergraduates and postgraduate students. At the present time the University of Gondar Comprehensive Specialized Hospital (UOGCSH) is the teaching and referral hospital in Gondar city and serves for an estimated of five million people of the communityin Northwest Ethiopia by expanding its research areas, outreaches, team training program (TTP) services. The actual data collection period was from January to March 2020.

All regular undergraduate and postgraduate students at UOGCMHS were the source and all students who were presented at the time of data collection period were study participants. All regular undergraduate medical and health science students who were registered in UOGCMHS were included in the study and participants who were Optometry department, and severally ill to take the screening were excluded from the study.

### Sample size determination and sampling technique

The sample size was determined using single population proportion formula by taking the following assumptions;

$$\text{n}={\text{Z}}^{2}\times \left(\text{P}\right)\times \left(\text{1}-\text{P}\right)/{\text{d}}^{2}$$

Where n = minimum required sample size, Z = value of z statistic at 95% confidence level = 1.96, P = proportion of visual impairment from previous study = 0.268 [18], d = maximum acceptable sampling error = 3.4%.


$${\left(1.96\right)}^{2}\times \left(0.268\right)\times \left(1-0.268\right)/{\left(0.034\right)}^{2}=654$$

**Fig 1:**
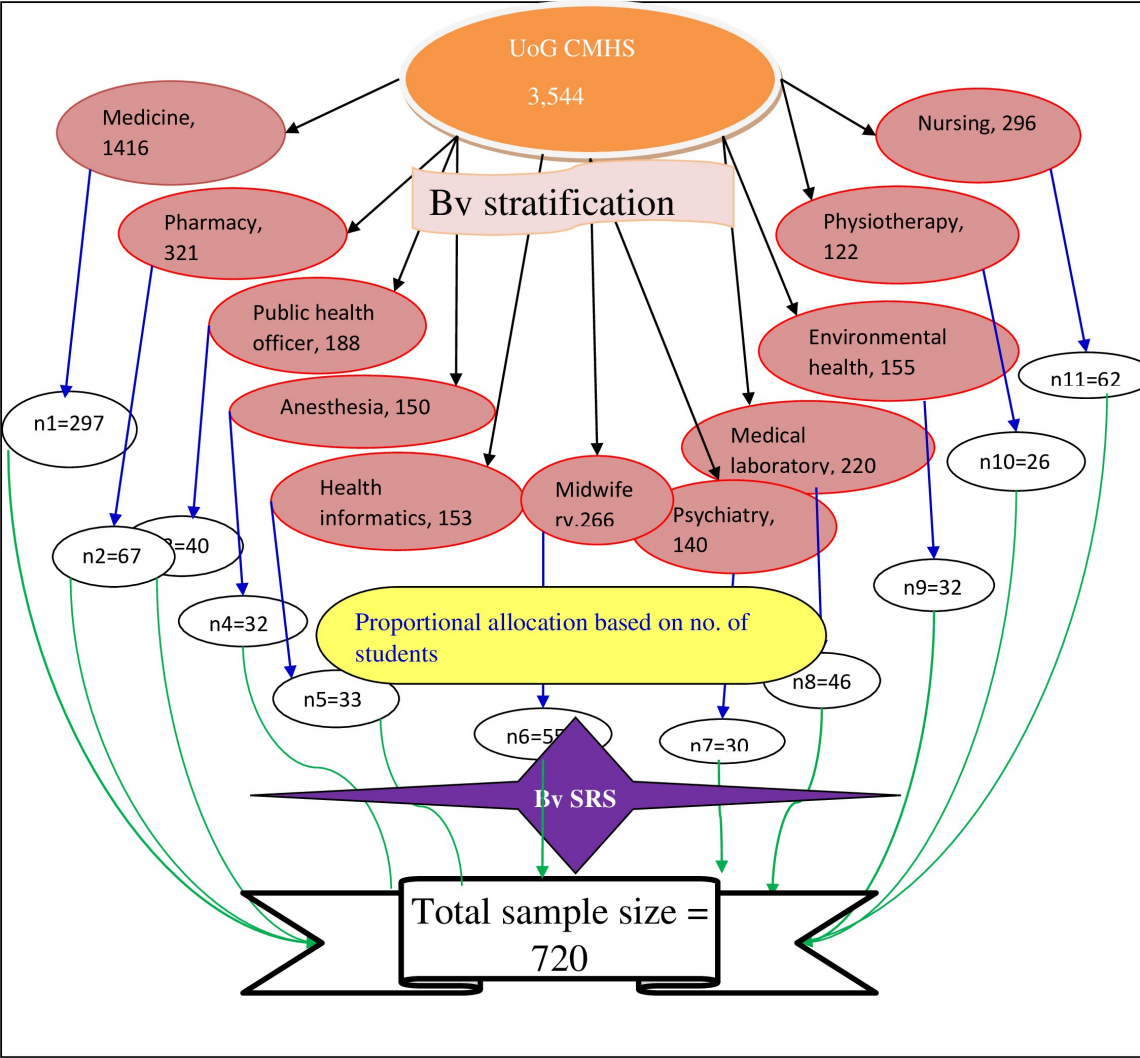
Sampling procedure for assessing visual impairment among medical and health science students at University of Gondar College of Medicine and Health Sciences.

By considering the above assumptions the minimum sample size was 654 and adding 10% non-response rate, the total sample size became 720. The study participants were selected from 11 departments within the college and those departments are stratified into batches. Systematic random sampling technique was used to select a total of 720 study participants were taken from a total of 3,544 medical and health science students. The students’ list was obtained from the registrar office and proportional numbers of individuals were taken from each batch. Sampling fraction was calculated for each department, and lottery method was applied to select the first student for screening from 1 to 5, and the next participants were selected by adding the fraction/interval k = 5 for each department. When the selected student were not found during the study next students were included after the student were double checked for his/her absenteeism (Fig 1).

### Data collection procedure

The questionnaire was adapted from different previously published articles [22–27]. The sociodemographic characteristics, history of neurological disorders, metabolic disorders, medication history and behavioral factors of study participants were collected by self-administered questionnaire. Five data collectors (three BSc Optometrists, two BSc Public Health Officers) and two supervisors trained by the principal investigator were participated in the data collection process. After the division of departments for the purpose of data collection the data collectors approach the participants based on the calculated interval (k) for each department. Study participants filled the questionnaire after having short clarification on common medical terms. The screenings of presenting visual acuity (VA) of participants were done by a standardized Snellen acuity chart at a distance of 6 meters from the participant in a well-illuminated room by Optometrists. Pinhole VA was taken also for those participants who had a presenting acuity of 6/12 or worse. Participants were diagnosed as having visual impairment and blindness when the presenting VA was < 6/12 and < 3/60 in the better eye respectively. Participants who were diagnosed as visually impaired and blind were given a referral paper to Gondar university Specialized Hospital eye center for further investigation and management options.

### Variables of the study

The outcome variable of the study was Visual impairment and the independent variables includes: Sociodemographic variables: (Age, sex, occupation, ethnicity, residence, income), Medical related factors: (Neurological disorders, metabolic disorders (DM, Anemia), history of eye disease and medication history), Behavioral and environmental factors: (Alcohol consumption, cigarette smoking, pesticide exposure and flashlight exposure).

### Operational definitions

**Visual impairment**: is a decrease in the ability of the eye to see shapes and the details of objects at a given distance and have different categories based on severity (Table 1) [4].

**Table 1:** Categories of severity of visual impairment according to the International statistical classification of diseases.

Category	Presenting distance VA in the better eye
**Normal vision**	**6/6**
**Mild VI**	**< 6/12**
**Moderate VI**	**< 6/18**
**Sever VI**	**< 6/60**
**Blindness**	**< 3/60**

**Medication history:** anyone who takes drugs like anti-psychotics/anti-depressants/anti-hypertensive/non-steroidal anti-inflammatory drugs [28,29].
**Substance use:** use of at least one of the substances (alcohol, cigarettes) in an individual’s life time.
**Current user:** a person who consumed any substance at least once within the last 30 days.
**Ever use**: use of any of the substances at least once in an individual’s life time.
**Pesticide exposure:** the duration, the frequency and negative effects of the active substance that comes in contact with the eye, skin or both, can be:
  **Danger**: Corneal opacity is not reversible within 7 days (eye), corrosive (skin)
  **Warning**: Irritation persisting for 7 days (eye), severe irritation at 72 hours (skin)
  **Caution**: Irritation reversible within 7 days or no irritation (eye), Moderate, Mild or slight irritation at 72 hours duration (skin) [30].
**Flashlight exposure:** the occupational exposure of radiation that is reflected from metal welding, Smartphone and Computers [31,32].

### Statistical analysis

The completed, clean and coded data were entered into Epi data version 3.1 and exported to Stata version 14 for formal analysis. Chi-square test was used to examine the difference between categorical variables. The summary statistics were presented with a mean (standard deviation), frequency (percentage), tables, and graphs. The relationship of each independent variable on each other was tested with a multicollinearity test and evidence of good fitting was checked with the Hosmer-Lemeshow goodness of fit test (p-value = 0.5406). A binary logistic regression statistical model was fitted between sociodemographic characteristics, history of neurological disorders, metabolic disorders, medication history and behavioral factors of study participants and visual impairment. All variables which have a p-value of less than 0.2 in bivariable logistic regression were included in multivariable logistic regression. Finally, the strength of statistical association between independent variables and the outcome variable (VI) was indicated by odds ratio (OR) with a 95% confidence interval (CI), and variables having a p-value of less than 0.05 were taken as statistically significant.

### Data quality management

The data were collected using well prepared English version of questionnaires after reviewing different literature and consultation of experienced experts in the subject area. To assure data quality, data collectors were trained for two days about how to use a pre designed form, how to perform a procedure and handle study participants. Pre-test was done on 36 individuals at Othonial College in Gondar city. A constant monitoring was also part of this study and was framed as an integral part of the data collection processes. The supervisors handled problems which arose, and received and checked the questionnaire for completeness in order to clean up the incorrect reporting and screening. The data were re-entered to check whether there is any inconsistency of data and to avoid any problem through the data entry processes. The test procedures were handled by trained professionals.

### Ethical consideration

Ethical clearance was obtained from University of Gondar Institutional Review Board, School of Medicine, College of Medicine and Health Sciences, with reference number of 1845/02/2020. Permission letter was also obtained from University of Gondar College of medicine and health science students’ associated dean office. The data were collected after written informed consent had been obtained from the study participants. Confidentiality, accountability and academic honesty were maintained throughout all phases of the research activities of the study and participants with an abnormal result were linked to the department of Optometry for consultation and to have further confirmatory eye examination.

## Results

### Socio-demographic, behavioral and environmental characteristics

In this study 654 participants were included with a response rate of 91.00%. The median age of participants was 23(SD±4) years. Four-hundred and thirty-nine (67.13%) students were Orthodox Christianity followers, and 513(78.44%) were from Amhara ethnic group. Less than half 266 (40.67%) participants were from rural areas. Among the study participants, 255 (39.00%) had a lifetime history of drinking alcohol, and 235 (35.93%) students were current alcohol drinkers (Table 2).

**Table 2:** Socio-demographic, behavioral and environmental characteristics of the study participants, College of Medicine and Health Sciences, University of Gondar, Northwest Ethiopia, 2020 (n = 654).

Variables	Categories	Frequency	Percent (%)
**Age in years**	≤ 25	533	81.50
	>25	121	18.50
**Sex**	Male	371	56.73
	Female	283	43.27
**Religion**	Orthodox	439	67.13
	Muslim	107	16.36
	Protestant	84	12.84
	Others^»^	24	3.67
**Ethnicity**	Amhara	513	78.44
	Oromo	52	7.95
	Qimant	38	5.81
	Others*	52	7.80
**Family occupation**	Farmer	261	39.91
	Merchant	88	13.46
	Governmental	227	34.71
	Non-governmental	78	11.93
**Family residence**	Urban	388	59.33
	Rural	266	40.67
**Alcohol consumption**	Current drinker	235	35.93
	Ever drink	20	3.07
	Never drink	399	61.01
**Amount of alcohol (liter per week)**	One	111	47.23
	Two	66	28.09
	Three and above	58	24.68
**Cigarette smoke**	Current smoker	5	0.76
	Ever smoke	12	1.83
	Never smoke	637	97.40
**Number of Cigarette (in pics)/day**	Less than five	3	60.00
	Five to ten	2	40.00
**Pesticide exposure**	Danger	3	0.46
	Warning	9	1.38
	Caution	30	4.59
	No	612	93.58
**Exposure of flash light**	Yes	62	9.48
	No	592	90.52

Others^»^ = Catholic, Adventist, Others* = Guraghe, Tigre.

### Clinical characteristics of participants

Two hundred and thirty-seven (36.24%) participants had history of migraine headache, whereas 66 (10.10%) had history of head trauma, and 49 (7.49%) of study participants had history of previous medication/drug use (Table 3).

**Table 3:** Clinical characteristics of the study participants, College of Medicine and Health Science, University of Gondar, Ethiopia, 2020 (n = 654).

Variables	Categories	Frequency	Percent (%)
**Any history of ocular problem**	Yes	148	22.63
	No	506	77.37
**Event of migraine/headache**	Yes	237	36.24
	No	417	63.76
**Event of head trauma/injury**	Yes	72	11.09
	No	582	88.91
**Presence of diabetic mellitus**	Yes	6	0.92
	No	648	99.08
**Family history of visual impairment**	Yes	65	9.94
	No	589	90.06
**History of medication/drug use**	Yes	49	7.49
	No	605	92.51
**Types of drugs**	Anti-depressant	8	16.32
	Anti-psychotic	24	48.98
	Anti-inflammatory	13	26.53
	Anti-hypertensive	4	8.16

### Prevalence of visual impairment

**Fig 2:**
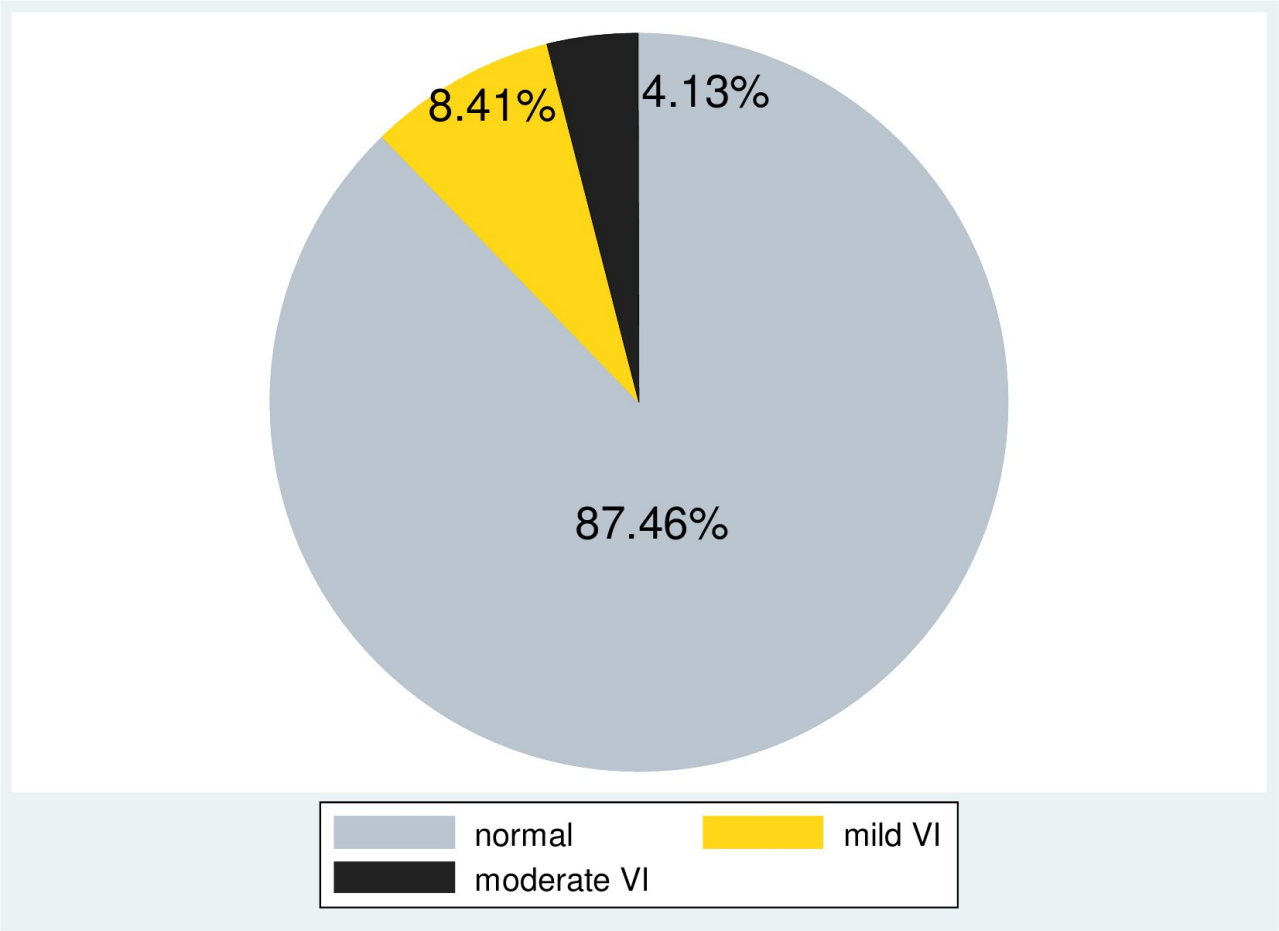
Distribution visual impairment among medical and health science, University of Gondar, Northwest Ethiopia, 2020.

The prevalence of VI was 12.50% (95% CI: (10.21–15.31%)). Among visual impairment students, 55 (67.07%) had mild visual impairment, and 27 (32.93%) had moderate visual impairment (Fig 2). Female participants had a higher prevalence of VI i.e. 36 (12.72%) than male, but not statistically significant. The prevalence of VI was higher among participants who had frequent history of head trauma 19 (26.39%) and family history of VI 22 (33.85%) (Table 4).

**Table 4:** Factors associated with visual impairment among medical and health science students in bivariable and multivariable logistic regression analyses, University of Gondar, Northwest Ethiopia, 2020.

Variables		VI		COR [95% CI]	AOR [95% CI]	P-value
		Yes n (%)	No n (%)			
**Age**	≤ 25	59 (11.07)	474 (88.93)	1		
	> 25	21 (17.36)	100 (82.64)	1.69(0.98, 2.90)	1.8 (1.02, 3.26)*	**0.041**
**Sex**	Male	46 (12.40)	325 (87.60)	1		
	Female	36 (12.72)	247 (87.28)	1.08(0.7, 1.73)	1.08 (0.64, 1.81)	0.088
**Year of study**	6^th^	7 (17.50)	33 (82.50)	1	1	
	2^nd^	28(16.77)	139(83.23)	0.94(0.38,2.36)	1.18(0.85, 2.93)	0.056
	3^rd^	19 (8.23)	212(91.77)	0.42(0.16,1.08)	0.5 (0.17, 1.45)	0.256
	4^th^	14(12.28)	100(87.72)	0.66(0.25,1.77)	0.9 (0.30, 2.81)	0.140
	5^th^	12(11.76)	90 (88.24)	0.63(0.23,1.73)	0.65 (0.21, 2.01)	0.426
**Alcohol Consumption**	Drink before 30 days	3 (13.04)	20 (86.96)	1.5 (0.44, 5.47)	2.8 (0.75, 10.70)	0.108
	Current drink	42 (17.87)	193(82.13)	2.2 (1.39, 3.63)	2.9 (1.70, 5.00)*	**0.002**
	Never drink	35 (8.84)	361 (91.16)	1	1	
**Head trauma/brain injury**	Yes	12 (18.18)	54 (81.82)	0.59 (0.3,1.60)	0.55 (0.26, 1.16)	0.316
	No	68 (11.56)	520 (88.44)	1	1	
**Family history of VI**	Yes	22 (33.85)	43 (66.15)	0.21(0.12,0.38)	0.16 (0.08, 0.31)	0.062
	No	58 (9.85)	531 (90.15)	1	**1**	

* = variables significant at p-value < 0.05, AOR: Adjusted Odd Ratio, COR: Crude Odd Ratio.

### Associated factors of VI among medical and health science students

Six variables that were associated with visual impairment in bivariable analysis (p<0.2), were entered into a multivariable logistic regression model. In the multivariable logistic regression, four variables were excluded. Accordingly, participants with the age of above 25 years had 1.8 times (AOR: 1.8, 95%CI: 1.02, 3.26), high odds of VI as compared to those ≤ 25 years old. The odd of VI among candidates with current alcohol consumption was 2.9 times (AOR: 2.9, 95%CI: 1.7, 5.00), higher than those without any history to use it (Table 4).

## Discussion

This study was conducted to determine the prevalence of visual impairment and to identify its associated factors among undergraduate medical and health science students. Hence, the study focused on the assessment of visual acuity to enable those affected individuals to follow better strategies that would overcome the consequences related to the disorder. To the best of our knowledge, this is the first study on VI among medical and health science students in Ethiopia.

The current study revealed that the magnitude of VI among medicine and health science students was 12.5% (95% CI: 10.21, 15.31%), which is comparable with a study done on adults in China (15.2%) [33], and Saudi Arabia (13.9%) [34], (14.90%) [35], respectively. However, the finding of this study was higher than the prevalence reported in Ghana 5.8% [36], Nigeria (5.02%) [37], (3.5%) [38], USA (7.5%) [39], Malaysia (8.9%) [40] and china (2.7%) [41]. But lower than the finding of the study in Gondar 26.8% [18], Debre Berhan (16.8%) [42], Nigeria 19% [43], Egypt (23.9%) [44], Zimbabwe 56.8% [45], Pakistan (26.7%) [46], United Arab Emirates and Lebanon (18.3%) [47] and England (17%) [48]. The possible reasons for this difference might be due to the difference in the study population, sample size, variation in geographical area, ethnicity.

In the present study, age and current alcohol drinking, were significantly associated with VI. Students aged greater than 25 years of age had 1.8 times higher odds of developing visual impairment compared with their counterpart. This is in agreement with the study done on school children in Ethiopia [49], and in Pakistan [46]. The possible reason for the increase in VI with age might be as age increases, there is an increase in environmental exposure and related changes in the sensitivity of the visual pathway [50]. These biological changes give rise to visual dysfunction of the eye and reduced visual acuity [49].

Furthermore, in our study, participants who were current alcohol drinkers had 2.9 times odds of developing VI. This is in line with a study done in the USA Austin Texas [51], Atlanta [52], and in Lexington at University of Kentucky [53]. This may be due to alcohol induced brain impairment as shown by neuro-imaging evidence appears that early brain changes result from alcohol drinking and if not resolved, these preventable and potentially reversible deficits may be progressive to loss of vision [24], and in a study on experiment using rats, Sancho-Tello and colleagues found chronic alcohol consumption impaired vision by its effect on the retina and retinal function via oxidative stress [52].

## Conclusion

The prevalence of VI among medical and health science students was relatively higher 12.50%. Age of students and current alcohol consumption were significantly associated with visual impairment. It is recommended that additional studies should be done to determine the magnitude and severity of VI by using better tools to accurately test visual acuity, an Ophthalmoscope, Tonometer and Tono-pen, which are coasty and not available to us.

Institutional leaders should take a responsibility to possess a well-programmed schedule for screening and creating awareness about visual impairment, and students who are enrolled in to higher education be screened regularly, not to discourage them from taking the course, but to make them conscious of the kind and status of their visual defect. If they have any problem, they should seek confirmatory examination.

Finally, we recommend the coming researchers to give emphasis on the challenges of visual impairment in the class room, practical sessions and overall quality of life of students.

### Limitation of the study

The limitation of the current study was recall-bias of the respondents to memorize the previous types of medication. The other limitation of this study is that the study used only Snellen acuity screening which only diagnose the status of current visual status of the participants. It is difficult to suggest the possible causes of VI impairment unless further investigation and refraction is done properly.

## Supporting information

S1 File Confidentiality and informed consent statement. (DOCX)

S2 File English version of data collection check list. (DOCX)

S3 File Amharic version of data collection check list. (DOCX)

## Abbreviations

AOR: Adjusted Odds Ratio
CI: Confidence Interval
COR: Crude Odds Ratio
UOGCMHS: University of Gondar College of medicine and health science
VI: Visual Impairment
