# Supplementary material for: Visual impairment and its associated factors among medical and health sciences students at the University of Gondar, Northwest Ethiopia
Source: PLoS One. 2021 Aug 19;16(8):e0255369. doi: 10.1371/journal.pone.0255369 (PMC8376000; doi:10.1371/journal.pone.0255369)
Supplement: S1 File — (DOCX) [file pone.0255369.s001.docx]

**Appendix I: Written consent form**

My name is …………….. I come here as data collector to assess visual impairment among Medical and Health Science students at University of Gondar College of Medicine and Health Science. I am going to give relevant information before you decide to be part of the research. If there is any word not clear for you when I give information stop and ask me to explain the question clearly. On this questionnaire your name will not be written and I am going to ask some questions related to sociodemography, health related issues. Participating and not participating is the full right of you. There is no risk or direct benefit in participating in this research project. However, your honest answer to these questions will help to focus on how to care on this problem in order to develop better strategies and solve the problems for the future. We would greatly appreciate your truthful and active participation in responding to this questionnaire. Put “X” at the box provided to show their willingness or unwillingness

A. Yes

B. No

If Yes continue the data collection process continue

Date of interview………………………….

Interviewer name………………………….

Signature ----------------------------------------

**አባሪ 1- የጽሑፍ ስምምነት ቅጽ**

ስሜ ……………. ……….ነው ፡፡ በጎንደር ዩኒቨርስቲ ህክምና እና ጤና ሳይንስ ኮሌጅ በሕክምና እና ጤና ሳይንስ ተማሪዎች መካከል ያለውን የእይታ ችግር (ቪዥዋል ኢምፓየርመንት) ለመገምገም እንደ ዳታ ሰብሳቢ ሆኜ ነው የመጣሁት ፡፡ የምርምር አካል ለመሆን ከመወሰንዎ በፊት ተገቢ መረጃ እሰጣለሁ ፡፡ መረጃን ስሰጥ ለእርስዎ ግልፅ ያልሆነ ቃል ካለ አቁሜ ጥያቄውን በግልጽ እንዳስረደዎ ይጠይቁ ፡፡ በዚህ መጠይቅ ላይ ስምዎ አይጻፍም እናም ከሶሾዲሞግራፊ ፣ ከጤና ጋር የተያያዙ ጉዳዮችን የሚመለከቱ አንዳንድ ጥያቄዎችን እጠይቃለሁ ፡፡ መሳተፍ እና አለመሳተፍ የእናንተ ሙሉ መብት ነው ፡፡ በዚህ የምርምር ፕሮጀክት ውስጥ መሳተፍ አደጋ ወይም ቀጥተኛ ጥቅም የለውም ፡፡ ሆኖም ለእነዚህ ጥያቄዎች በሐቀኝነት የሰጡት መልስ የተሻለ ስትራቴጂዎችን ለማዳበር እና ለወደፊቱ ችግሮቹን ለመፍታት በዚህ ችግር ላይ ለማተኮር ይረዳል ፡፡ ለዚህ መጠይቅ መልስ ለመስጠት በእውነተኛ እና ንቁ ተሳትፎዎ በጣም እናደንቃለን ፡፡ ፈቃደኛነታቸውን ወይም ፈቃደኛ አለመሆናቸውን ለማሳየት በተጠቀሰው ሳጥን ላይ “X” ን ያድርጉ

ሀ. አዎ

ለ. አይደለም

አዎ ከሆነ የመረጃ አሰባሰቡ ሂደት ይቀጥላል

የቃለ መጠይቅ ቀን ……………………..

የቃለ መጠይቅ ስም ……………………..

ፊርማ ----------------------------------------
