# Supplementary material for: Visual impairment and its associated factors among medical and health sciences students at the University of Gondar, Northwest Ethiopia
Source: PLoS One. 2021 Aug 19;16(8):e0255369. doi: 10.1371/journal.pone.0255369 (PMC8376000; doi:10.1371/journal.pone.0255369)
Supplement: S2 File — (DOCX) [file pone.0255369.s002.docx]

**English version questioner**

1. Sociodemographic factors (circle for closed and write for open questions)

| S/N | Variables |  |
| --- | --- | --- |
| 101 | Age | ___________________ |
| 102 | Sex | 1. Male 2. Female |
| 103 | Religion | 1. Orthodox 2. Muslim 3. Protestant 4. Catholic 5. Others_________ |
| 104 | Ethnicity | 1. Amhara 2. Oromo 3.Tigre 4.Guraghe 5.others |
| 105 | Occupation(family) | 1. Farmer 2. Merchant 3. Government employee 4. Non government employee 5. Others__________ |
| 106 | Residence (family) | 1. Urban 2. Rural |

1. Behavioral and Environmental factors (circle the alternative for closed and write for open questions)

| S/N | Questions | Answer | If no, skip to Question |
| --- | --- | --- | --- |
| 201 | Have you ever used any substance(alcohol,cigarate) in your life time? | 1. Yes 2. No |  |
| 202 | Have you ever drunk alcohol in your life time? | 1. Yes 2. No | Qs **205** |
| 203 | If yes , have you drink alcohol within the last 30days? | 1. Yes 2.No |  |
| 204 | If yes to Q203, how many bottles of beer/liquor you drink per week? | _____ |  |
| 205 | Have you ever smoked cigarette in your life time? | 1. Yes 2. No | Qs**208** |
| 206 | If yes , are you currently smoking cigarette (within the last 30days? | 1. Yes 2.No |  |
| 207 | If yes to Q206, how many cigarettes you smoke daily (in pcs) | ________ |  |
| 208 | Have you any exposure to pesticides | 1.Yes 2. No | **Qs 214** |
| 209 | If yes, route of exposure | 1. Eye 2. Dermal  3. Both |  |
| 210 | If through Eye, duration of the effect | 1. Less than 7days 2. 7 days  3.More than 7days |  |
| 211 | Severity of the effect | 1.Corneal opacity  2. Irritation  3. No irritation |  |
| 212 | If dermal, duration of the effect | 1.less than 72hrs 2. 72hrs  3. More than 72hrs |  |
| 213 | Severity of the effect | 1.Corrosive  2.Sever irritation 3.Moderat irritation  4.Slight irritation |  |
| 214 | Have you occupational exposure to Flash light, in metal work (welding) | 1.Yes 2.No |  |

1. Clinical correlated factors (circle the alternative for closed and write for open questions)

| 301 | Any history of ocular problem | 1. Eye disease (trachoma, cataract,……  2. Eye trauma  3. Eye surgery  4. Photophobia 4. Others _______________ |
| --- | --- | --- |
| 302 | Event of migraine / severe headache( frequent pain aggravate by light, noise, and smells) | 1. Yes 2. No |
| 303 | Episode of head trauma / accident | 1. Yes 2. No |
| 304 | Presence of diabetic mellitus | 1. Yes 2. No |
| 305 | Occasion of Light headedness, Tiredness, Jaundice, bleeding | 1.Yes 2.No |
| 306 | Previous medication / drug use | 1. Yes  2. No |
| 307 | If yes, which one | 1. Anti-depressant  2. Anti-psychotic  3. Anti-inflammatory  4. Anti-Hypertensive 6. Others___________ |

1. Standardize visual examination using Snellen’s Chart test

| 401 | Visual acuity | Score | Result in the better eye  _________ |
| --- | --- | --- | --- |
|  |  | Right eye_________________ |  |
|  |  | Left eye__________________ |  |
