# Supplementary material for: Visual impairment and its associated factors among medical and health sciences students at the University of Gondar, Northwest Ethiopia
Source: PLoS One. 2021 Aug 19;16(8):e0255369. doi: 10.1371/journal.pone.0255369 (PMC8376000; doi:10.1371/journal.pone.0255369)
Supplement: S3 File — (DOCX) [file pone.0255369.s003.docx]

**የአማርኛ መጠይቅ**

1. ሶሽዮሞግራፊክ ምክንያቶች (ለተዘጋው አማራጩን ክበብ እና ለተከፈቱ ጥያቄዎች ይጻፉ)

| ቁጥር | ተለዋዋጮች |  |
| --- | --- | --- |
| 101 | ዕድሜ | ___________________ |
| 102 | ፆታ | 1. ወንድ 2. ሴት |
| 103 | ሃይማኖት | 1. ኦርቶዶክስ 2. ሙስሊም 3. ፕሮቴስታንት 4. ካቶሊክ 5. ሌሎች_________ |
| 104 | ዘር | 1. አማራ 2. ኦሮሞ 3. ትግሬ 4.ጉራጌ 5. ቅማንት 6. ሌሎች |
| 105 | ሥራ (ከቤተሰብ ጋር) | 1. ገበሬ 2. ነጋዴ 3. የመንግሥት ሠራተኛ 4. የግል ሠራተኛ የሆነ  5. ሌሎች__________ |
| 106 | መኖሪያ (ከቤተሰብ ጋር) | 1. ከተማ 2. ገጠር |

1. ስነ ምግባራዊ እና አካባቢያዊ ምክንያቶች (ለተዘጋው አማራጩን ክበብ እና ለተከፈቱ ጥያቄዎች ይፃፉ)

| ቁጥር | ጥያቄዎች | መልስ | ካልሆነ, ፣ ወደ ጥያቄ ይዝለሉ |
| --- | --- | --- | --- |
| 201 | በሕይወትዎ ውስጥ ማንኛውንም ንጥረ ነገር (አልኮሆል ፣ ሲጋራራ) ተጠቅመው ያውቃሉ? | 1. አዎ 2. አይደለም |  |
| 202 | በሕይወትዎ ውስጥ አልኮል ጠጥተው ያውቃሉ? | 1. አዎ 2. አይደለም | ቁ **205** |
| 203 | አዎ ከሆነ ባለፉት 30 ቀናት ውስጥ አልኮል ጠጥተዋል? | 1. አዎ 2. አይደለም |  |
| 204 | ለ ቁ 203 አዎ ከሆነ በሳምንት ስንት ጠርሙስ አልኮል መጠጥ ይጠጣሉ? | _____ |  |
| 205 | በህይወትዎ ውስጥ ሲጋራ በጭስ መቼም ቢሆን አጭሱ ያውቃሉ? | 1. አዎ 2. አይደለም | ቁ **208** |
| 206 | አዎ ከሆነ በአሁኑ ጊዜ ሲጋራ እያጨሱ ነው (በመጨረሻዎቹ 30 ቀናት ውስጥ? | 1. አዎ 2. አይደለም |  |
| 207 | ለ ቁ 206 አዎ ከሆነ በየቀኑ ስንት ሲጋራ ያጨሳሉ (በፍሬ) | ________ |  |
| 208 | ለፀረ-ተባይ መድሃኒቶች ምንም ዓይነት ተጋላጭነት አለዎት | 1. አዎ 2. አይደለም | **ቁ 214** |
| 209 | አዎ ከሆነ የተጋላጭነት መንገድ | 1. በአይን  2. በቆዳ  3. ሁለቱም |  |
| 210 | በአይን በኩል ከሆነ የውጤቱ ቆይታ | 1. ከ 7 ቀን በታች  2. 7 ቀናት  3. ከ 7 ቀናት በላይ |  |
| 211 | የውጤት ከባድነት | 1. የኮርኔል ግልጽነት  2. ማቃጠል  3. ማቃጠል የለም |  |
| 212 | የቆዳ ከሆነ ፣ የውጤቱ ቆይታ | 1.ከ72ሰዓታትያልበለጠ  2. 72 ሰዓቶች  3. ከ 72 ሰዓቶች በላይ |  |
| 213 | የውጤቱ ከባድነት | 1. በጣም ከፍተኛ ማቃጠል  2. ከፍተኛ ማቃጠል  3. መካከለኛ ማቃጠል  4. ትንሽ ማቃጠል |  |
| 214 | በብረት ሥራ (ብየዳ) ውስጥ ለፍላሽ መብራት ተጋላጭነት አለዎት | 1. አዎ 2. አይደለም |  |

1. ክሊኒካዊ ተያያዥነት ያላቸው ምክንያቶች (ለተዘጋው አማራጩን ክብ ያድርጉ እና ለተከፈቱ ጥያቄዎች ይጻፉ)

| 301 | ከአሁን በፊት ማንኛውም የዓይን ችግር ታሪክ አለ | 1. የአይን በሽታ (ትራኮማ ፣ የአይን ሞራ ፣ ……  2. የአይን ጉዳት  3. የዓይን ቀዶ ጥገና  4. የብርሃን መፍራት 4. ሌሎች ------------------------ |
| --- | --- | --- |
| 302 | የጭንቅላት ራስ ምታት ክስተት (ተደጋጋሚ ህመም በብርሃን ፣ በጩኸት እና በመሽተት ያባብሳል) አለ | 1. አዎ 2. የለም |
| 303 | የጭንቅላት አደጋ / ስብራት አጋጥሞዎት ያቃል | 1. አዎ 2. የለም |
| 304 | የስኩዋር በሽታ አለብዎት | 1. አዎ 2. የለም |
| 305 | የጭንቅላት መቅለል ፣ የድካም ስሜት ፣ የጃንሲስ ፣ የደም መፍሰሱ አጋጣሚ አለ | 1. አዎ 2. የለም |
| 306 | ከዚህ በፊት የተጠቀሙት መድኃኒት አለ | 1. አዎ 2. አይደለም |
| 307 | አዎ ከሆነ ፣ የትኛው የትኛው | 1. ፀረ-ድብርት  2. ፀረ-ሳይኮቲክ (ለጭንቀት)  3. ፀረ-ብግነት (ለእብጠት)  4. ፀረ-ግፊት (ለደም ግፊት) 6. ሌሎች___________ |

1. የስኔሌን ሙከራ በመጠቀም የእይታ ምርመራን መደበኛ ያድርጉ (ውጤት ፃፍ)

| 401 | የማየት ችሎታ | የውጤት | የተሻላዉ አይን ዉጤት  ------------- |
| --- | --- | --- | --- |
|  |  | የቀኝ ዐይን _________________ |  |
|  |  | የግራ ዐይን__________________ |  |
